# Absence of the DNA repair enzyme human 8-oxoguanine glycosylase is associated with an aggressive breast cancer phenotype

**DOI:** 10.1038/bjc.2011.518

**Published:** 2011-11-22

**Authors:** P Karihtala, S Kauppila, U Puistola, A Jukkola-Vuorinen

**Affiliations:** 1 Department of Oncology and Radiotherapy, Oulu University Hospital and University of Oulu, P.O. Box 22, FIN-90029, Oulu, Finland; 2 Department of Pathology, Oulu University Hospital and University of Oulu, P.O. Box 50, FIN-90029, Oulu, Finland; 3 Department of Obstetrics and Gynecology, Oulu University Hospital and University of Oulu, P.O. Box 24, Oulu, Finland

**Keywords:** 8-oxodG, human 8-oxoguanine DNA glycosylase, oxidative stress, reactive oxygen species

## Abstract

**Background:**

8-Oxo-7,8-dihydro-2′-deoxyguanosine (8-oxodG) is the most abundant marker of DNA damage and it reflects oxidative stress. Human 8-oxoguanine glycosylase (hOGG1) is a DNA-repair enzyme that participates in 8-oxodG removal.

**Methods:**

hOGG1 protein expression was immunohistochemically studied in 96 patients with local or locally advanced breast cancer and in 20 lesions of non-malignant breast disease. 8-OxodG levels had been previously determined in all patients.

**Results:**

hOGG1 was overexpressed in invasive *vs* non-invasive lesions (*P*=0.006). 8-OxodG and hOGG1 had a significant inverse association (*P*=0.046). Lack of hOGG1 expression was associated with the most poor prognostic factors of breast cancer. In addition, all triple-negative breast carcinomas (TNBCs) were hOGG1 negative (*P*=0.027 *vs* non-TNBCs). Patients with a lack of both hOGG1- and 8-oxodG immunostaining showed extremely poor breast cancer-specific survival compared with those with either 8-oxodG- or hOGG1-positive tumours (*P*<0.000005).

**Conclusion:**

The current results imply that absence of hOGG1 expression is associated with features of aggressive breast cancer. Tumours lacking both 8-oxodG and hOGG1 seem to indicate especially poor prognosis.

Enhanced generation of reactive oxygen species (ROS) and consequent oxidative stress are characteristic features of malignant tumours (Karihtala and Puistola, 2011). The most widely used marker of oxidative stress is 8-oxo-7,8-dihydro-2′-deoxyguanosine (8-oxodG) and this adduct is considered to reflect ROS-derived damage in DNA (Wiseman and Halliwell, 1996).

8-OxodG is a potent threat to genomic integrity and therefore there are several mechanisms to prevent its accumulation. In frontline defense, antioxidant enzymes are able to reduce levels of ROS before their interaction with DNA. Multiple, highly conserved DNA repair mechanisms exist in aerobic organisms, and they partly overlap (Evans *et al*, 2004; Hirano, 2008). Human 8-oxoguanine DNA glycosylase (hOGG1) cleaves 8-oxoGua from DNA and the adduct is excreted to the bloodstream and finally to urine.

We have previously demonstrated that 8-oxodG is paradoxically substantially present in breast carcinomas in patients with good prognosis, and serum 8-oxodG levels are also higher in patients with biologically less aggressive breast cancer (Sova *et al*, 2010; Karihtala *et al*, 2011a). Expression of 8-oxodG is also significantly diminished in invasive breast carcinomas when set against hyperplasias and ductal carcinoma *in situ* (DCIS) (Karihtala *et al*, 2011b). However, this is in contrast to other oxidative stress markers, which show more explicably increased expression in invasive breast carcinomas compared with non-invasive lesions (Karihtala *et al*, 2011b). We hypothesised that the reason behind these apparently paradoxical results may lie in induction of the enzyme hOGG1 in breast carcinomas, although assessment of hOGG1was unavailable at that time. Therefore, in this study, we used hOGG1 immunostaining in stage I–III breast carcinomas to test this hypothesis. We also tested whether hOGG1 is associated with prognosis or clinicopathological prognostic factors such as steroid receptor expression, proliferation, tumor size, nodal status, HER2 status and triple-negative phenotype (triple-negative breast carcinoma (TNBC)).

## Materials and methods

### Samples

The study material consisted of 116 formalin-fixed, paraffin-embedded breast tumor samples. In all, 96 of the samples were invasive carcinomas from patients with local or locally advanced breast cancer and 20 samples were classified as atypical ductal hyperplasia (ADH) (*n*=15) or ductal carcinoma *in situ* (*n*=5) (Tavassoli and Devilee, 2003). The tissue samples were fixed in neutral formalin, embedded in paraffin blocks and stored at the Department of Pathology, Oulu University Hospital, and they dated from the years 2003–2006.

### Immunohistochemistry

hOGG1 immunohistochemical analysis was carried out using the same prospective series, as follows. Three and half micron-thick sections were cut from a representative paraffin block and placed on SuperFrostPlus glass slides (Menzel-Gläser, Braunschweig, Germany). The sections were first de-paraffinized in xylene and rehydrated in a descending series of ethanol concentrations, neutralised from endogenous peroxidase using Peroxidase Block and incubated with the Protein Block. The sections were incubated overnight at room temperature with rabbit polyclonal anti-hOGG1 (NB 100-106, Lot F4, Novus Biologicals, Littleton, CO, USA) diluted 1 : 500 in antibody diluent (S2022, Dako, Glostrup, Denmark). After washing with phosphate-buffered saline (PBS) the slides were incubated with Post Primary Block and then, after washes, incubated using the NovoLink Polymer Detection System (RE7150-K, Leica Microsystems, Wetzlar, Germany) for 30 min. After rinsing in distilled water, the Dako Envision peroxidase detection system (Dako K5007) was used and the sections were then counterstained with haematoxylin and finally mounted with Immu-Mount (Shandon, Pittsburgh, PA, USA). Negative controls were prepared using the same procedure except that the primary hOGG1 antibody was replaced with PBS or serum isotype controls (Zymed Laboratories Inc., South San Francisco, CA, USA).

8-OxodG immunostaining of this material from the same patients has been reported previously (Sova *et al*, 2010). The staining procedure was mainly same as with hOGG1. In brief, a primary antibody dilution 1 : 125 (clone N45.1, Gentaur, Kampenhout, Belgium) was used overnight at +4°C. Secondary antibody was from Dakopatts (Glostrup, Germany) and aminoethyl carbazole (Zymed Laboratories Inc.) was used as a chromogen.

The hOGG1 immunostaining results were divided semiquantitatively into four groups: −= no immunostaining present; +=weak immunostaining (5–20% positive cells); ++=moderate immunostaining (21–80% positive cells); +++=strong immunostaining (>80% positive cells). The cutoff for steroid receptor positivity was >9% and for Ki-67 the cutoffs were as follows: negative <5% +=5–14% ++=15–30% +++ >30%. The expression of HER2 was also studied by means of immunohistochemistry and when there was a HER2-positive result (either +, ++ or +++ on a scale of 0 to +++), gene amplification status was determined using chromogenic *in situ* hybridisation. Grading and tumor size were divided into the following subgroups: grade I–II group, grade III group, T1 group and T2–4 group.

### Statistical analysis

SPSS 17.0 for Windows (IBM, Chicago, IL, USA) was used for statistical analysis. The significance of associations was determined by using Fisher's exact two-sided test and the Mann–Whitney test. Survival was analysed by means of Kaplan–Meier curves and the log-rank test. Cox multivariate regression analysis was used for multivariate analysis. In survival analysis, only confirmed death caused by breast cancer was considered as an event. Statistical significance was set at *P*<0.05.

## Results

**Figure 1:**
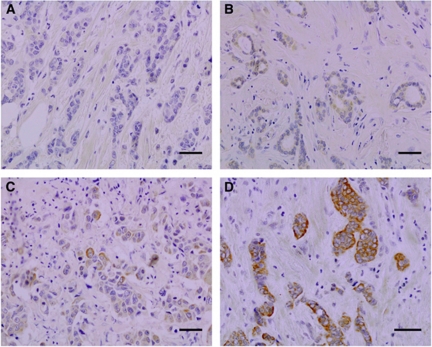
hOGG1 immunoreactivity scored as number of positive cells and staining intensity in invasive ductal breast carcinoma. Spectrum of typical hOGG1 staining pattern: (**A**) negative, (**B**) weak, (**C**) moderate and (**D**) strong. Positive staining signals are seen as brown in malignant epithelial structures. The scale bars represent 0.05 mm.

Expression of hOGG1 was virtually entirely cytoplasmic; only sporadic-positive nuclei were observed (Figure 1). We could not observe hOGG1 expression in the stroma of invasive carcinoma. Staining positivity was confined to the malignant epithelium. hOGG1 staining positivity in benign epithelial structures was rather indistinct, generally negative. A few benign epithelial structures showed barely discernible weak staining positivity (− to +). Distribution of immunostaining results is shown in Table 1. When negative/weak immunostaining results were compared with moderate/strong results in the pre-invasive and invasive lesions, invasive breast carcinomas showed significant hOGG1 overexpression (*P*=0.0064). Likewise, when hOGG1 expression was classified as either negative/weak or moderate/strong, there was an inverse association with 8-oxodG immunostaining (*P*=0.046).

In invasive breast carcinomas, negative hOGG1 immunostaining was associated with several factors linked traditionally to poor prognosis: very high Ki-67 expression (*P*=0.023), grade III differentiation (*P*=0.01), presence of lymphatic vessel invasion (*P*=0.036), and absence of oestrogen (*P*=0.016) and progesterone (*P*=0.016) receptor expression. Carcinomas with a triple-negative phenotype (lack of HER2, and oestrogen and progesterone receptors; *n*=6) showed absence of hOGG1 expression, whereas 47 of 90 (52.2%) non-TNBCs showed hOGG1 expression (*P*=0.027). hOGG1-positive immunostaining was also connected with non-ductal histology (*P*=0.037).

**Figure 2:**
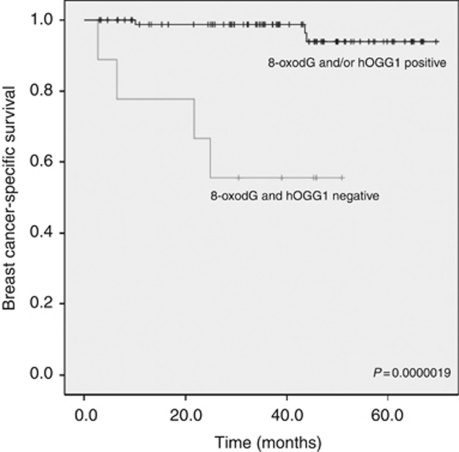
Kaplan–Meier curve showing breast cancer-specific survival rates when both 8-oxodG- and hOGG1-negative cases are compared with other tumours.

Women with both 8-oxodG-negative and hOGG1-negative results showed significantly decreased breast cancer-specific survival compared with those patients with at least one positive marker (log-rank test, *P*=0.0000019) (Figure 2). The former group had a mean survival time of 34.5 months (95% CI: 21.8–47.2 months) and the latter 67.5 months (95% CI: 65.3–69.7 months). This was also an independent prognostic factor in Cox regression analysis when compared with traditional clinicopathological factors. hOGG1-negative (irrespective of 8-oxodG status) breast cancer samples showed a nonsignificant trend to predict poor prognosis (*P*=0.066).

## Discussion

8-OxodG base excision repair enzymes are found from plants to primates (Hirano, 2008) and the importance of 8-oxodG removal is highlighted in knock-out experiments, where hOGG1^−/−^ mice show accumulation of 8-oxodG in their genomes and susceptibility to development of at least lung tumours, ulcerative colitis-induced colorectal adenocarcinomas and UV-induced skin tumours (Sakumi *et al*, 2003; Kunisada *et al*, 2005; Liao *et al*, 2008). The hOGG1 protein exists as two isoforms: *α*-hOGG1 and *β*-hOGG1. The latter is present solely in mitochondria, although its biological function is still unclear (Hashiguchi *et al*, 2004; Hirano, 2008). *α*-hOGG1 seems to be responsible for both mitochondrial and nuclear DNA repair, which has been demonstrated in human cell lines (Hashiguchi *et al*, 2004). Mitochondrial hOGG1 may also function as a chaperone protein to prevent oxidative stress-mediated mitochondrial toxicity (Panduri *et al*, 2009). Leakage from the mitochondrial electron transport chain is the main source of ROS, at least under physiological conditions, and there is more metabolic damage in mitochondria compared with nuclei (Richter *et al*, 1988; Turrens, 1997).

As far as we know, hOGG1 protein expression has not been studied in breast cancer *in vivo*. In the current study 49% of invasive cancers showed hOGG1 expression, which is roughly comparable to the figure of 62% reported previously in head and neck cancer, although a different grading system was used in that study (Fan *et al*, 2001). We found localisation to be virtually entirely cytoplasmic, most probably mitochondrial, which in line with several previous observations in which hOGG1 immunohistochemical expression has been assessed (Salim *et al*, 2008; Ku *et al*, 2009). Cytoplasmic hOGG1 expression most probably reflects mitochondrial hOGG1 expression, but to discover whether it is the *α* or *β* isoform, requires studies with different methods.

Despite the limited number of pre-invasive breast lesions (ADH or DCIS) in our material, they showed notably less hOGG1 expression than the invasive breast carcinomas, only one case showed moderate or strong immunostaining, compared with nearly a third of the invasive lesions. It is reasonable to assume that there is no need for intensive DNA-repair enzyme induction until oxidative stress levels become considerable in invasive cancers. Invasive breast carcinomas without the protective effect of hOGG1 are clearly associated with aggressive features of breast carcinomas, including high grade, increased proliferation, lymphatic vessel invasion and steroid receptor-negative disease. hOGG1 is induced not only under oxidative conditions, but interestingly also by BRCA1 protein, deficiency of which is common in TNBCs (Mabley *et al*, 2005; Saha *et al*, 2010). Although our material included only six TNBC cases, none of them expressed hOGG1, whereas the majority of the non-TNBCs showed hOGG1 expression. It therefore appears that in addition to other defects in base excision repair of TNBC tumours (Foulkes *et al*, 2010), these tumours also have impaired hOGG1 function.

Although there was an association between hOGG1 expression and aggressive carcinomas, hOGG1 did not alone predict breast cancer-specific survival significantly. 8-OxodG-positive tumours showed significantly less hOGG1 expression, which is biologically reasonable. One of the most significant observations in this study was the discovery of a subgroup of patients with extremely poor prognosis. We have previously reported that tissue 8-oxodG levels are greatly reduced in invasive breast cancers compared with premalignant lesions and also that the absence of 8-oxodG is an independent prognostic factor of poor prognosis in breast carcinomas (Sova *et al*, 2010; Karihtala *et al*, 2011a, 2011b). The ultimate reason behind this remains unclear, although this result is now convincingly confirmed in different patient materials and setups. When we combined patients with both 8-oxodG- and hOGG1-negative tumours, we found a subgroup of patients where 45.4% died of breast cancer within 2 years of surgery. Confirming studies are obviously required, but the observed difference is independent of other traditional prognostic factors and clinically highly significant.

Based on the results above, we conclude that hOGG1 protein is overexpressed in invasive breast carcinomas compared with premalignant lesions and in invasive disease lack of hOGG1 expression is associated with an aggressive phenotype. Absence of both 8-oxodG and hOGG1 may be a combination to find women with extremely poor prognosis, although further studies are required.

## Figures and Tables

**Table 1:** The distribution of hOGG1 immunostaining in non-invasive and invasive breast carcinomas

**OGG1 immunostaining**	**ADH/DCIS (%)**	**Invasive breast carcinomas (%)**
−	12 (60.0)	49 (51.0)
+	7 (35.0)	13 (13.5)
++	0 (0.0)	19 (19.8)
+++	1 (5.0)	15 (15.8)

Abbreviations: ADH=atypical ductal hyperplasia; DCIS=ductal carcinoma *in situ*; hOGG1=human 8-oxoguanine glycosylase.
